# Fenretinide: A Novel Treatment for Endometrial Cancer

**DOI:** 10.1371/journal.pone.0110410

**Published:** 2014-10-23

**Authors:** Navdha Mittal, Saurabh Malpani, Matthew Dyson, Masanori Ono, John S. Coon, Julie J. Kim, Julian C. Schink, Serdar E. Bulun, Mary Ellen Pavone

**Affiliations:** 1 Division of Reproductive Biology, Department of Obstetrics and Gynecology, Feinberg School of Medicine at Northwestern University, Chicago, Illinois, United States of America; 2 Division of Reproductive Endocrinology & Infertility, Department of Obstetrics and Gynecology, Feinberg School of Medicine at Northwestern University, Chicago, Illinois, United States of America; 3 Division of Gynecology Oncology, Department of Obstetrics and Gynecology, Feinberg School of Medicine at Northwestern University, Chicago, Illinois, United States of America; 4 Department of Obstetrics and Gynecology, Spectrum Health Medical Group, Grand Rapids, Michigan, United States of America; Wayne State University School of Medicine, United States of America

## Abstract

Resistance to progestin treatment is a major hurdle in the treatment of advanced and reoccurring endometrial cancer. Fenretinide is a synthetic retinoid that has been evaluated in clinical trials as a cancer therapeutic and chemo-preventive agent. Fenretinide has been established to be cytotoxic to many kinds of cancer cells. In the present study, we demonstrate that fenretinide decreased cell viability and induced apoptosis in Ishikawa cells, which are an endometrial cancer cell line, in dose dependent manner *in-vitro*. This effect *was found to be* independent of retinoic acid nuclear receptor signaling pathway. *Further*, we have shown that this induction of apoptosis by fenretinide may be caused by increased retinol uptake via STRA6. Silencing of STRA6 was shown to decrease apoptosis which was inhibited by knockdown of STRA6 expression in Ishikawa cells. Results of an *in-vivo* study demonstrated that intraperitoneal injections of fenretinide in endometrial cancer tumors (created using Ishikawa cells) in mice inhibited tumor growth effectively. Immunohistochemistry of mice tumors showed a decrease in Ki67 expression and an increase in cleaved caspase-3 staining after fenretinide treatment when compared to vehicle treated mice. Collectively, our results are the first to establish the efficacy of fenretinide as an antitumor agent for endometrial cancer both *in-vitro* and *in-vivo*, providing a valuable rationale for initiating more preclinical studies and clinical trials using fenretinide for the treatment of endometrial cancer.

## Introduction

Endometrial cancer is the most prevalent gynecologic cancer in the United States, with an estimated 49,560 new cases and 8,190 - deaths occurring in 2013 [1]. The probability of a female being diagnosed with this cancer during her lifetime is about one in 38 [2]. The incidence of this reproductive organ malignancy has increased by an average of 1.1% per year from 2004 to 2008 and death rates have also increased by an average of 0.3% per year from 1998 to 2007 [3]. Surgery, chemotherapy and radiation therapy are three main established approaches for the treatment of endometrial carcinoma [4], [5]. Surgical removal of the uterus and adjuvant therapy result in a greater than 90% of five year survival rate. Recurrence due to resistance to chemotherapy or radiotherapy presents another major challenge for healthcare professionals. Therefore, it is of utmost important to identify new and effective treatments for endometrial cancers.

Clinically, progestins, including megace (megestrol acetate) have been used to treat endometrial malignancies because of the antagonistic effect of progesterone on estrogen action. Progestins are used as primary agents to treat advanced or recurrent disease, those who are poor candidates for surgery and in younger women who would like to preserve their future fertility. A complete response can be achieved when progestin therapy is used in women with endometrial hyperplasia and well-differentiated endometrial adenocarcinoma; however, the effectiveness of progestin therapy drops with an increase in disease severity. Additionally, recurrences can occur once progesterone therapy is stopped [6], [7].

In search of targeted therapeutic agent against endometrial cancer, previous *in-vitro* studies from our lab demonstrated marked inhibition of proliferation of endometrial cancer Ishikawa cell line by retinoic acid (RA) and the RA agonist AM580 compound [8]. However, their clinical usage has been, thus far, limited by their unfavorable side effects profile [9]. RA and their derivatives (either natural or synthetic compounds) have a recognized role in the regulation of cell growth, differentiation and apoptosis. RA has the potential for the treatment and prevention of cancers [10], [11]. Retinol (the dominant form of retinoids in the human body) must be converted into retinoic acid to show its biological activity. The primary source of RA is dietary vitamin A, which is taken up in the intestine and packaged as retinyl esters in the liver. These retinyl esters are secreted into the circulation bound to retinol binding protein (RBP) and subsequently taken into cells via *STRA6* (Stimulated by RA 6), a crucial cell surface receptor for RBP [12]. Previously, our laboratory has shown that STRA6 is the principal regulator of retinol uptake in the endometrium and that the decreased expression of this gene in endometriosis can contribute to decreased hydroxysteroid (17-beta) dehydrogenase 2 (HSD17β2) mRNA expression [13], leading to persistently elevated levels of estradiol. We have also shown that retinoids decrease estrogen production by inducing HSD17β2 expression in endometrial Ishikawa cells [14].

STRA6 is the main cell-surface receptor responsible for retinol uptake. Once inside the cell, retinol can be oxidized to the more biologically active RA by alcohol dehydrogenases. RA is then directed from the cytoplasm to specific nuclear hormone RA receptors (RARs), and the retinoid X receptors (RXRs) by two intra-cytoplasmic carrier proteins, specifically cellular RA binding protein 2 (CRABP2) and fatty acid binding protein 5 (FABP5) [15]. Finally, the unused RA is metabolized and disposed out of the cells by CYP26 family of enzymes [16].

Fenretinide [N-4-hydroxyphenyl retinamide (4-HPR)], a synthetic derivative of all-trans retinoic acid has the capability to initiate cell apoptosis even in ATRA-resistant cell lines with the added benefit of having a minor side-effects profile. Human studies have found that the major side effects include diminished adaptation to darkness of the eyes, skin and mucosal dryness, pruritus, urticaria, gastrointestinal discomfort and alteration to ocular surfaces. These side effects were relatively frequent but mild. As such, it is emerging as one of the most promising antitumor agents [17]. Studies have demonstrated that fenretinide can induce cytotoxicity in multiple human cancer cell lines *in vitro* [18]–[23]. Clinically, fenretinide has been used as both a chemopreventive agent in breast [24], bladder [25] and oral mucosal cancers [26], and as a chemotherapeutic agent in pediatric [27], [28] and adult cancers [29]–[31]. The mechanism of action of fenretinide induced cell death has not yet been completely elucidated. However, it has been proposed that its inhibitory effects may be mediated by both retinoic acid receptor-dependent and -independent mechanisms [17].

The present study investigated the therapeutic potential of fenretinide as an antitumor agent as well as its potential mechanism of action against endometrial cancer both *in-vitro* and *in-vivo.*

## Materials and Methods

### Ethics statement

All the animal experiments were approved by Northwestern University Animal Care and Use Committee.

### Endometrial cancer cells culture

Endometrial epithelial Ishikawa cell lines (a kind gift from Dr. Masato Nishida Kasumigaura National Hospital, Tsuchiura, Ibaraki, Japan) were derived from human malignant endometrial epithelial cells [32]. Ishikawa cells were cultured in monolayers at 37°C, 5% CO2 incubator in a mixture of DMEM and F12 (1∶1) medium with 5% fetal bovine serum (FBS), 1% sodium pyruvate and 1% penicillin-streptomycin, hygromycin antibiotics solution (Life Technologies, Grand Island, New York).

### Small interfering RNA (siRNA) knockdown

Ishikawa cells were grown to approximately 80% confluency, then transfected with a nontargeting negative control siRNA (siCTL) or siRNAs against STRA6 (Life Technologies) at 30 nM concentration using Lipofectamine RNAiMAX according to manufacturer's recommendations (Life Technologies). Transfected cells were treated with either DMSO or 6 µM fenretinide (Sigma-Aldrich, St. Louis, MO) solution 60 hours post transfection. Cells were collected after 24 hours of treatment and processed for real-time PCR or Western blotting.

### Cell viability assay

Prestoblue (Life Technologies) cell viability reagent was used to estimate the cell viability. Prestoblue is a resazurin - based membrane permeable solution that upon reduction, converts into resorufin, a red fluorescent compound which can be quantitatively measured to determine cell viability [33]. Ishikawa cells were seeded (5×10^3^ cells per well) in 96-well plates and cultured for 24 hours at 37°C. The cells were then serum starved for 16–18 hours [34], [35]. Cells were then grown in fresh medium and treated with different concentrations of fenretinide and megestrol acetate (Sigma-Aldrich) for 24 h at 37°C. The cytotoxicity of fenretinide and megestrol acetate was measured by adding the Prestoblue reagent according to the manufacturer's instructions. The fluorescence signal was measured to determine cell viability on the Synergy HT plate reader from Bio-Tek with the KC4 3.4 software at 530/590 nm.

### Western blot analysis

Ishikawa cells were treated with different concentrations of fenretinide and megestrol acetate (Sigma-Aldrich) or vehicle, lysed in RIPA [50 mM Tris, pH 8.0,150 mM sodium chloride, 1.0% Triton X-100, 0.5% sodium deoxycholate, 0.1% SDS (sodium dodecyl sulphate) supplemented with protease and phosphatase inhibitors (Sigma-Aldrich). Clear lysates were obtained after centrifugation at 14,000 rpm for 15 minutes, and the protein concentration was measured using the Micro BCA kit (Thermo Scientific, Rockford, Illinois). Proteins were resolved by sodium dodecyl sulfate–polyacrylamide gel electrophoresis on NOVEX NuPAGE 4-12% Bis-tris gels (Life Technologies) and transferred onto polyvinylidene difluoride membranes (Whatman, GE Healthcare Life Sciences, Piscataway, NJ). The membranes were blocked with 5% non-fat dry milk for one hour at room temperature and hybridized with specific primary antibodies for total and cleaved PARP [poly (ADP-ribose) polymerase], Caspase-9 & cleaved Caspase-9 (Cell Signaling Technology, Beverly, MA) overnight at 4°C. The protein bands were visualized using an enhanced chemiluminescence reagent ECL Super Signal West Femto detection kit (Thermo Scientific) after hybridization with a horseradish peroxidase-conjugated goat anti-rabbit secondary antibody (Cell Signaling Technology). The membranes were stripped with Restore Western Blot Stripping Buffer (Thermo Scientific) and probed with beta-actin (β-actin) antibody (Sigma-Aldrich) for protein loading control.

### Real time PCR

Total RNA was isolated from Ishikawa cells using Quiagen RNaeasy Kit (Quiagen, Valencia, CA) according to the manufacturer's protocol. The purity and concentration of extracted RNA were determined using the ND-1000 Spectrophotometer (NanoDrop). 1μg of RNA was reverse-transcribed with the Q-script cDNA super mix (Quanta Biosciences Inc., Gaithersburg, MD, USA) according to manufacturer's protocol. Real-time quantitative PCR was performed with the ABI 7900 Sequence Detection and the ABI Power Syber Green gene expression systems (Applied Biosystems, Foster City, CA, USA). The mRNA was quantified using commercially available specific primers to STRA6, CRBP1, CYP26A1, CRABP2, FABP5, RAR-α, RAR-β, and RXR-α (Quiagen) and the housekeeping gene GAPDH (Integrated DNA Technologies, Chicago, IL). The fold change in expression was calculated using the ΔΔCt method [36] with GAPDH as an internal control. PCRs were performed on an ABI PRISM 7000 Sequence Detection System (Applied Biosystems) for 40 cycles (95°C for 15 s, 60°C for 1 minute) after 10 minutes incubation at 95°C.

### Xenograft mouse model

Female CD-1 athymic nude mice (4–5 weeks old, Charles River Laboratories International Inc., Wilmington, MA) were maintained under specific pathogen-free conditions based on the guidelines established by the Northwestern University, Institutional Animal Care and Use Committee. Mice were ovariectomized, and, an estradiol pellet (1.7 mg/pellet, 60 day release) (Innovative Research of America, Sarasota, Florida) was implanted subcutaneously. Ishikawa human endometrial cancer cell-line cells (2×10^6^/0.1 ml) were suspended in 1∶1 PBS/Matrigel (BD Biosciences, San Jose, California) and injected subcutaneously into both right and left flanks of each mouse Tumors were allowed to grow and once the tumors reached 50–150 mm^3^ (which occurred in about 2 weeks), mice were divided into 4 groups: 1. vehicle, 2. megestrol acetate, 3 fenretinide (Tocris Bioscience, Bristol, UK) and Fenretinide + megestrol acetate. Megestrol actate pellets (21 mg, 21-day release for 1 mg/day) (Innovative Research of America) were implanted subcutaneously. The mice were given 120 mg/kg of fenretinide at a volume of 10 ml/kg of body weight, 5 times per week for 3 weeks via intraperitoneal injection. Control mice were similarly treated with i.p. injections of 5% ethanol in 0.9% sodium chloride solution containing 1.65 mg/ml of bovine serum albumin. There was no evidence of local or systemic toxicity after i.p. treatment with the dose administered [37] (Fig. S2). Mice were weighed and tumor sizes were measured with calipers twice a week throughout the entire course of treatment. The mice were euthanized using CO2 asphyxiation and the tumors were resected. Tumor tissues from mice were weighted, then embedded in a paraffin block and subjected to immunohistochemistry or hematoxylin and eosin (H&E) staining. Tumor volumes were calculated using the formula:
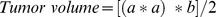



Where “a” is the shorter of two perpendicular axes [35].

### Immunohistochemistry

Tissues were fixed in formalin, paraffin embedded, and 4 µm tissue sections were cut. After de-paraffinization of tissue sections, heat induced antigen retrieval was performed in a 10 mM sodium citrate buffer (pH 6.0) with 0.05% Tween (Sigma) for 20 min in a pressure cooker. The slides were allowed to cool for 30 minutes at room temperature and were then washed in TBS-T for 5 minutes. The Dako EnVision HRP IHC kit was used (Dako North America, Inc., Carpinteria, CA). Endogenous peroxidase activity was blocked with Hydrogen peroxide (3%) for 10 minutes followed by 2 washes of TBS-T for 10 minutes. For Ki67 immunolabelling, non-specific binding sites were blocked with protein block, and the tissue sections were then incubated with Ki67 primary antibody (Dako) overnight at 4°C in a humidified chamber. Anti- mouse secondary antibody was then applied to the tissue sections for 1 hour at room temperature and then washed in TBS-T twice for 5 minutes. For cleaved caspase-3, non-specific proteins were blocked using 5% normal donkey serum, incubated with cleaved Caspase-3 (Cell Signaling Technology) overnight, then an ABC kit protocol (Vector Laboratories Inc., Burlingame, CA) was followed. DAB solution was used to develop color, and Mayer's Hematoxylin was used to counterstain the sections. The 28% ammonium hydroxide was used as a bluing reagent. The sections were dehydrated via 2 changes of 95% ethanol, 2 changes of 100% ethanol, and 3 changes of Xylene, and then mounted onto cover slips using Cytoseal XYL mounting media (Richard-Allan Scientific). The slides were visualized using Zeiss upright AXIO TissueFAXS version 3.5.5120.120 scope (TissueGnostics GmbH, Vienna, Austria).

### Statistical analysis

All quantitative data are expressed as mean values ± standard error, and significant differences were determined by one-way ANOVA using GraphPad Prism6. A probability value of

*P*<0.05 was used as the criterion for statistical significance.

## Results

### Fenretinide decreases cell viability and increases apoptosis of endometrial cancer Ishikawa cells *in-vitro*

**Figure 1:**
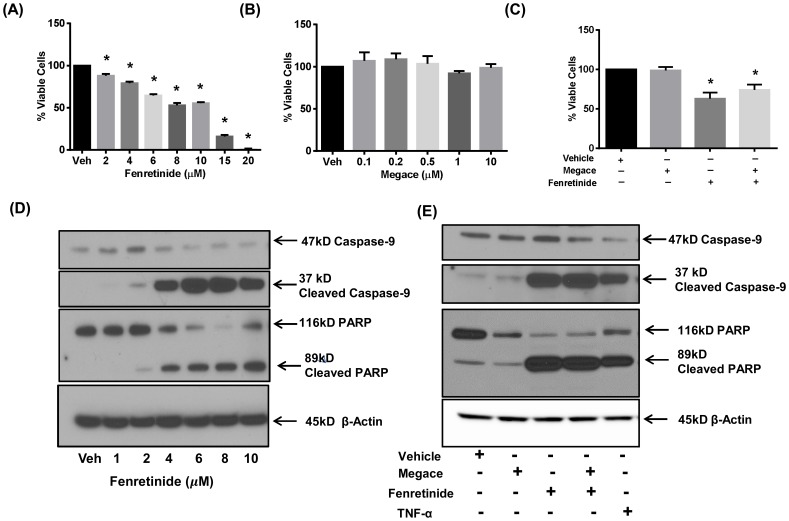
Effects of Fenretinide on Ishikawa cell viability and apoptosis (A–E). Ishikawa cells were treated with DMSO (Veh), (**A**) 2–20μM fenretinide, (**B**) 0.1–10μM megace or (**C**) a combination of megace + fenretinide for 24 h and cell viability was measured using the Prestoblue reagent. Data are presented as fold change in relative fluorescence units from DMSO treated samples and represent the mean ±SEM of 4–5 independent experiments. An asterisk indicates a significant (*P<0.05) decrease in cell viability in Ishikawa cells treated with fenretinide as compared to the DMSO treated group. (**D**) Ishikawa cells were treated with DMSO (Veh) or 1–10μM fenretinide which induced apoptosis in a dose dependent manner within 24 h of treatment as demonstrated by increases in cleaved caspase-9 and cleaved-PARP protein levels by western blotting. (**E**) The treatment of Ishikawa cells with megace did not have any effect on these markers of apoptosis. Actin was used as loading control. Blots are representative of 3 independent experiments.

The treatment of Ishikawa cells with fenretinide significantly inhibited cell viability and increased apoptosis in a dose-dependent manner (Fig.1A & 1D). Fenretinide at 6 to 20 µM caused a decrease of 38% to 99% respectively in cell viability and increase in apoptosis as evident by increases in protein levels of cleaved caspase -9 and cleaved-PARP as compared to vehicle- treated cells. Fenretinide was found to be toxic to the cells at doses higher than 2 µM concentration after 24 hours of treatment (data not shown). In order to determine the effects of megace, Ishikawa cells were treated with megace alone at different concentrations and in combination with fenretinide for 24 hours and cell viability & apoptosis were assessed. Megace did not have any effect on Ishikawa cell viability and apoptosis up to 10 µM alone as well as in combination with 6 µM fenretinide (Fig.1B, 1C & 1E). These results suggest that the fenretinide reduces cell viability and induces apoptosis in Ishikawa cells *in-vitro* making it a promising candidate to test its efficacy *in-vivo*.

### Fenretinide exerts an increase in retinol uptake via STRA6 and CRBP1

**Figure 2:**
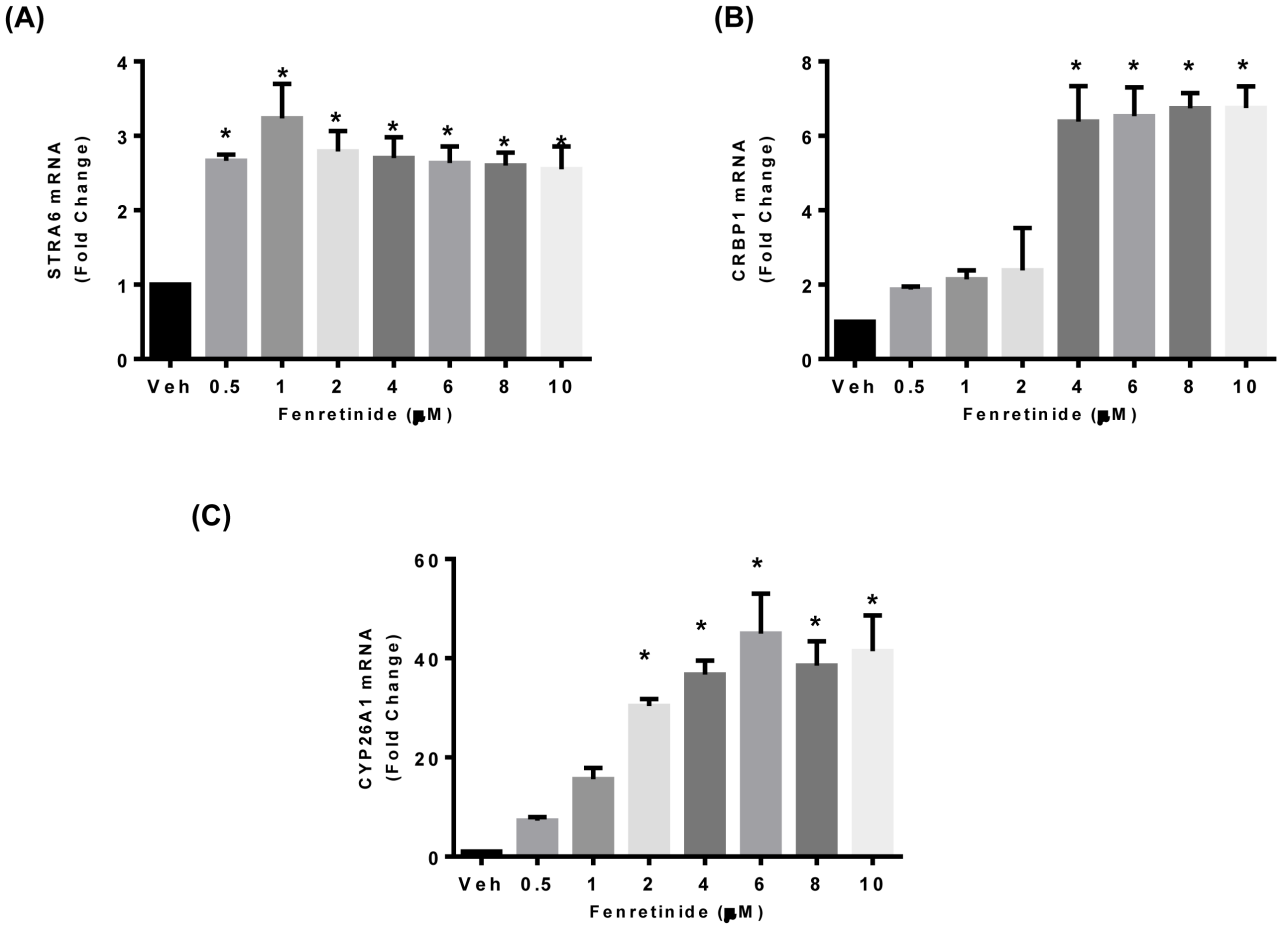
Fenretinide increases retinol uptake genes expression in endometrial cancer Ishikawa cells (A–C). Total RNA was isolated from Ishikawa cells treated with DMSO (Veh) or 0.25–10μM fenretinide for 24 h and the expression of genes involved in retinol uptake was measured by RT-qPCR. Data are presented as fold change in relative mRNA expression of fenretinide treated cells from DMSO treated samples cells and represent the mean ±SEM of 3 independent experiments. An asterisk indicates a significant (*P<0.05) increase in mRNA expression of (**A**) STRA6, (**B**) CRBP1 and (**C**) CYP26A1 genes in Ishikawa cells treated with fenretinide as compared to DMSO treated group.

**Figure 3:**
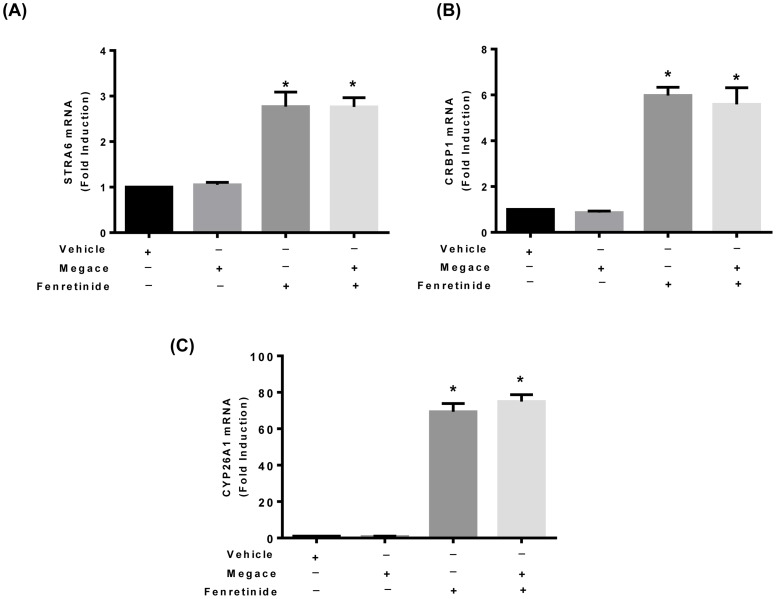
Effects of megace on retinol uptake genes in endometrial cancer Ishikawa cells (A–C). Total RNA was isolated from Ishikawa cells treated with DMSO (Vehicle), megace, fenretinide and a combination of megace and fenretinide both. Relative mRNA expressions of (**A**) STRA6, (**B**) CRBP1 and (**C**) CYP26A1 were analyzed with RT- qPCR. Data are presented as fold change in relative expressions of mRNA from DMSO treated samples and represent the mean ±SEM of 5 independent experiments. An asterisk indicates a significant (*P<0.05) increase in mRNA expression of these genes in Ishikawa cells treated with fenretinide or megace + fenretinide cells as compared to DMSO treated cells.

To determine the effect of fenretinide on the retinoic acid signaling pathway in endometrial cancer, real time RT PCR was performed. The mRNA levels of STRA6, CRBP1 and CYP26A1 significantly increased with the fenretinide treatment [Fig.2 (A–C)]. The mRNA of these genes remained unchanged with megace treatment alone or in combination with fenretinide [Fig.3 (A–C]. However, fenretinide did not have any effect on the mRNA levels of nuclear receptor genes (RAR-α, RAR-β, RXR- α), or the intra-cytoplasmic carrier proteins CRABP2 and FABP5 (Figure S1).

### STRA6 knockdown influences fenretinide mediated apoptosis in endometrial Ishikawa cells

**Figure 4:**
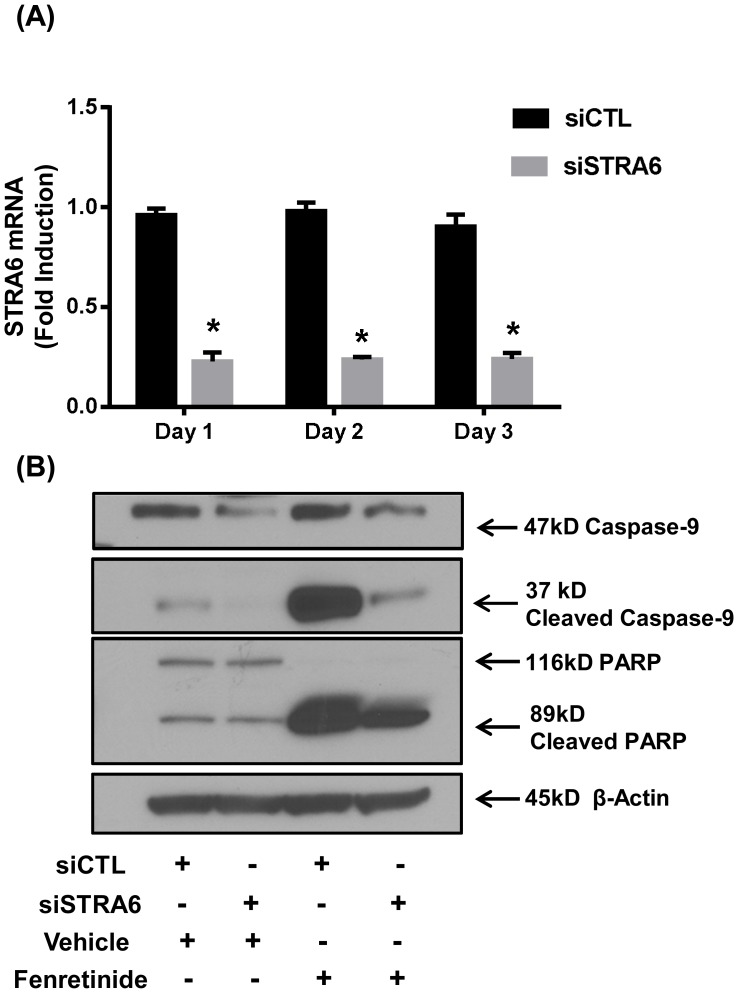
Inhibition of fenretinide induced apoptosis by knockdown of STRA6 gene expression. Ishikawa cells were transfected with 30 nM siCTL (nontargeting negative control siRNA) or siSTRA6. (**A**) Total RNA was isolated from untreated Ishikawa cells at 3 consecutive days of post transfection and the expression of STRA6 gene was measured by RT-qPCR. Data are presented as fold change in relative mRNA expression of STRA6 in siSTRA6 transfected cells and siCTL transfected cells and represent the mean ±SEM of 5 independent experiments. An asterisk indicates a significant (*P<0.05) decrease in mRNA expression of STRA6. (**B**) Transfected Ishikawa cells were treated with DMSO (Veh) or 6μM fenretinide for 24 h after and immunoblotting for apoptotic markers was performed. STRA6 gene expression knockdown inhibited fenretinide induced apoptosis in Ishikawa cells as demonstrated by decreases in protein levels of cleaved caspase-9 and cleaved-PARP. Blots are representative of 3 independent experiments.

STRA6 is the primary cell surface retinol receptor in the cells responsible for retinol uptake. To establish whether STRA6 is essential for fenretinide induced apoptosis in Ishikawa cells, endogenous STRA6 expression was knocked down using siRNA. As shown in Fig. 4A, we were able to significantly knockdown (76%) the expression of STRA6 in Ishikawa cells. Knockdown of STRA6 abrogates the fenretinide induced apoptosis in Ishikawa cells (Fig. 4B) as shown by diminished levels of cleaved caspase-9 and cleaved PARP when STRA6 is silenced. These results strongly support our hypothesis that STRA6 is crucial for fenretinide induced apoptosis in endometrial Ishikawa cells.

### Fenretinide inhibits tumor progression: a mouse xenograft model

**Figure 5:**
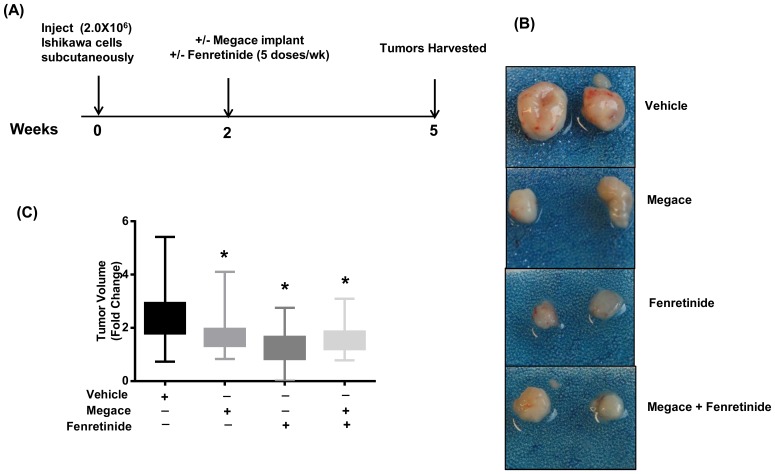
Effect of fenretinide on endometrial tumor progression in an Ishikawa xenograft mouse model (A–C). (**A**) Ovariectomized nude CD-1 female mice were injected with Ishikawa cells in both the flanks and estrogen pellets were implanted. Tumors were allowed to grow for 2 weeks. After 2 weeks of tumor establishment, the mice were treated with vehicle (20 mice), megace (19 mice), fenretinide (21 mice) or megace + fenretinide (20 mice) and tumors were harvested after 3 weeks of treatment. (**B**) Images of representative excised tumors from each group. (**C**) The fold change in tumor volume (from day 1 treatment to day of sacrifice) of vehicle, fenretinde, meagce or both fenretinide + megace treated mice was plotted after 3 weeks of treatment. Data are shown as means ± min to max of two different experiments. The P values indicate a significant inhibition by fenretinide, megace or megace + fenretinide both on tumor growth (*P<0.05). The largest decrease in tumor size was observed in the mice treated with fenretinide alone.

Initial *in-vitro* observations suggested that the anticancer activity of fenretinide may arise from its potential to promote apoptosis in tumor cells. To examine this antitumor activity of fenretinide *in-vivo*, the mice were engrafted with subcutaneous tumors. Fenretinide treatment significantly suppressed tumor progression in mice as compared to vehicle-treated mice (Figure 5B) based on fold change in tumor size. The decreases in tumor volume were also significant in megace treated as well as megace + fenretinide treated (Figure 5B) as compared to vehicle- treated mice. Moreover, mice seemed to tolerate fenretinide and megace treatments without apparent symptoms of toxicity or serious loss of body weight compared with vehicle-treated group (Figure S2).

### Treatment with fenretinide decreases proliferation and increases apoptosis in mice tumors

**Figure 6:**
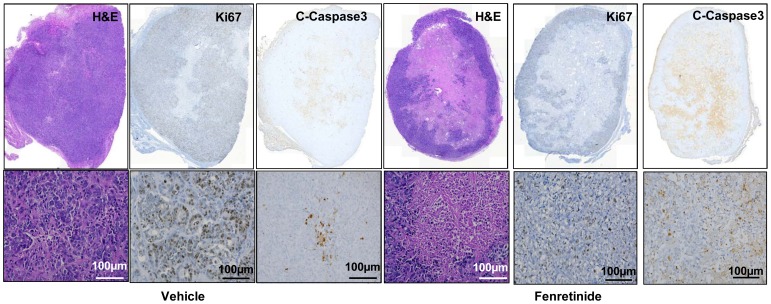
Inhibition of cell proliferation and increase in apoptosis following fenretinide treatment. Excised tumors were fixed in formalin, paraffin embedded and sectioned. (**A**) H & E staining and immunohistochemical staining with anti-Ki67 antibody (proliferation marker) and anti-cleaved caspase -3 (apoptosis marker) of whole sections of tumor xenografts. (**B**) The images are at 100*μ*m.

The effect of fenretinide on cell proliferation and apoptosis was evaluated by immunohistochemistry and H&E staining of Ishikawa endometrial tumor sections after 3 weeks of treatment. (Fig.6). Proliferation of the cells, as measured by Ki67 protein levels, decreased with fenretinide treatment. The levels of cleaved caspase-3 (an apoptosis marker), were negligible in the tumors of vehicle- treated mice, while more staining was evident in the tumors of fenretinide treated mice. Taken together, these data indicate that Ishikawa tumor development was suppressed by fenretinide through its ability to inhibit cell proliferation and increase in apoptosis of Ishikawa cells both *in-vitro* & *in-vivo*.

## Discussion

Fenretinide has already been shown to be an effective therapeutic and chemopreventive alternative in breast and ovarian cancers [17]. The present study demonstrates the use of fenretinide as a possible anti- tumor agent against endometrial cancer using both *in-vitro* and *in-vivo studies*. Fenretinide decreased human endometrial cancer Ishikawa cell viability by inducing apoptosis as demonstrated by an increase in cell death markers (cleaved caspase-9 and cleaved PARP). It also inhibited tumor growth *in-vivo*, as seen by a decrease in cell proliferation (Ki67) and an increase in apoptosis (Cleaved caspase-3) in mice tumor xenografts. Additionally, we have shown that the antitumor effects of fenretinide are mediated by an increase in retinol uptake, with an increased expression of STRA6 seen in fenretinide treated cells.

Recently, studies by Carrera et al have shown the role of STRA6 in p53 mediated cell death responses in normal and cancer cells, which is similar to our research findings and found to be independent of the downstream activation of RA targets [38]. They demonstrated that transfection with STRA6 induced a substantial amount of apoptosis in RA sensitive ovarian cancer cells (specifically PA-1 and A1847) as well as in RA insensitive HCT116 colon cancer cells. Additionally, the authors found that STRA6 transfection induced both a pro-apoptotic and tumor suppressive affect. These findings, together with the findings of our study, suggest that STRA6 induction may have a potential role in tumor suppression.

Previous literature suggests an interaction in the successful treatment of cancer cells with chemotherapeutic drugs and their potential to trigger apoptotic pathways. It is still not clear whether the biological effects of chemotherapeutic agents are mediated by a direct interaction with nuclear retinoid receptors or via novel nuclear receptor-independent pathways [39]. We found that treatment of Ishikawa cells with fenretinide resulted in cellular apoptosis via induction of Caspase-9, caspase -3 and PARP. The induction of these apoptotic pathways has also been seen in breast cancer [17] and ovarian cancer [40]. Interestingly, a phase III breast cancer prevention trial in premenopausal women has demonstrated that treatment with fenretinide decreased the incidence of second breast malignancies and ovarian cancers [41].

In contrast to our present study, other studies have demonstrated that RA analogues may also act by activating nuclear receptors. Specifically, Ishikawa cells proliferation was decreased and apoptosis was induced via RA signaling involving RAR/RXR activation [8].

Progestins have long been used in reproductive aged women who want to preserve their fertility, as well as in advanced and recurrent endometrial cancer cases which cannot be treated with anticancer agents, However, 30% or more patients with endometrial cancer exhibit resistance to hormonal therapies, limiting the use of progestin therapy [42]. In the present study, megace alone did not induce any apoptosis in our endometrial cancer model, nor did it potentiate the anti- tumor activity of fenretinide *in-vitro* or *in-vivo*.

The limitations of the present study are the use of single mouse model and the use of cultured endometrial Ishikawa cells instead of primary endometrial cancer cells. Using primary culture is difficult because of the inability to grow primary cancer cells without stromal factors. Further studies are warranted to establish antitumor capacity of fenretinide in different endometrial cancer cell lines and tumor mouse models.

In conclusion, we have demonstrated that fenretinide decreases cell viability, increases apoptosis, and causes a decrease in tumor size. We believe that fenretinide induced apoptosis because of an increase in retinol uptake, specifically by increasing gene expression of STRA6. We believe that this study is among the first to demonstrate that fenretinide has anti-tumor potential against endometrial cancer. We believe that future preclinical and clinical trials are needed to validate its potential as a new possible therapeutic candidate for the treatment of endometrial cancer.

## Supporting Information

Figure S1 **Treatment of Ishikawa cells with fenretinide at 0.25–10** *μ* **M concentrations for 24 h did not alter the expression of retinoic acid nuclear receptor genes.** The data is representative of means ± standard error of three different experiments.
(TIF)

Figure S2 **Treatments with either fenretinide or megace have no effect on mouse body weights.** The body weights were measured from Vehicle treated or drug treated groups of mice twice a week during the whole experiment rime.
(TIF)
