# Supplementary figures and images for: Fenretinide: A Novel Treatment for Endometrial Cancer
Source: PLoS One. 2014 Oct 23;9(10):e110410. doi: 10.1371/journal.pone.0110410 (PMC4207704; doi:10.1371/journal.pone.0110410)

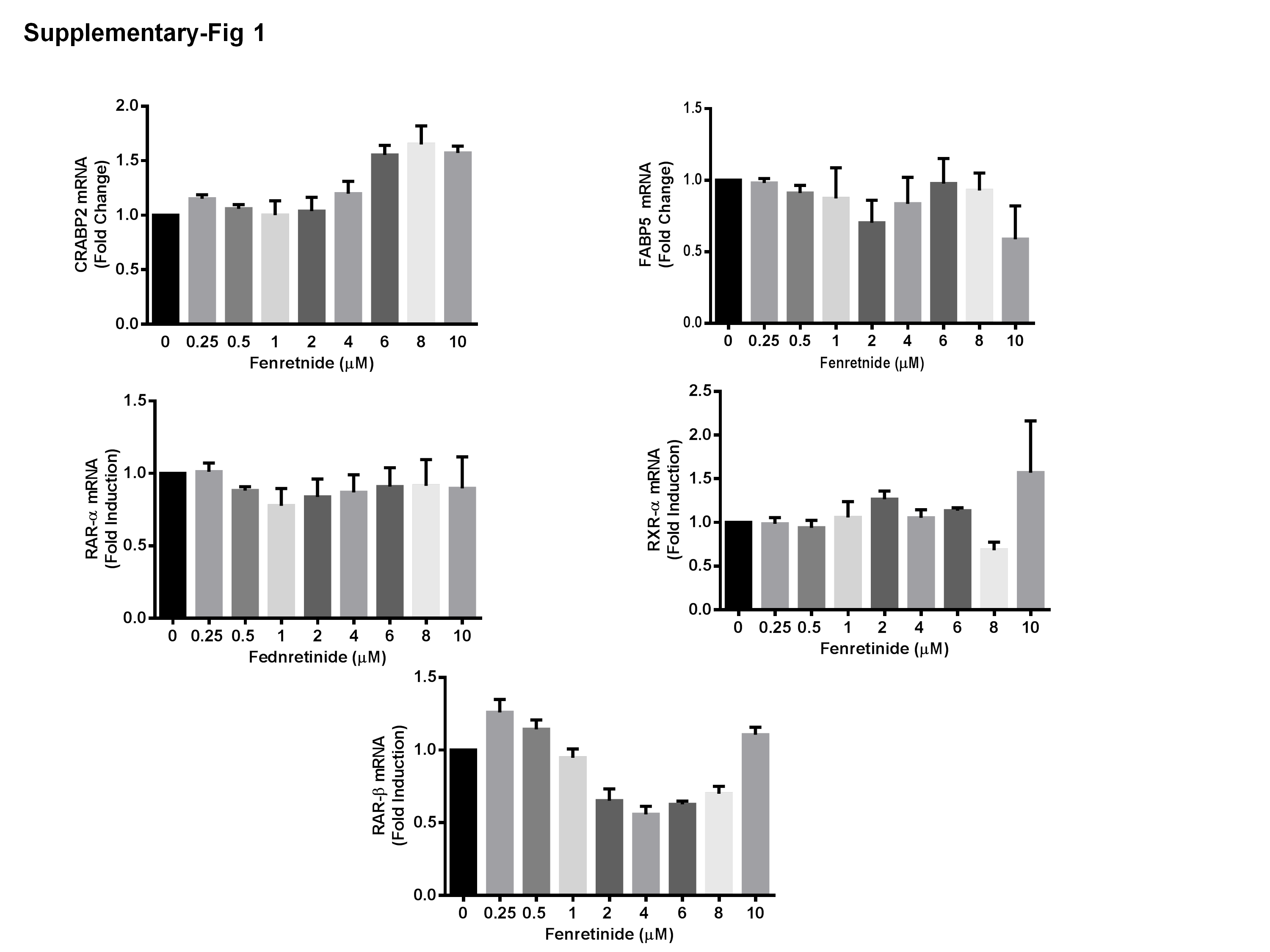

Supplement: Figure S1 — Treatment of Ishikawa cells with fenretinide at 0.25–10 μ M concentrations for 24 h did not alter the expression of retinoic acid nuclear receptor genes. The data is representative of means ± standard error of three different experiments. (TIF) [file pone.0110410.s001.tif]

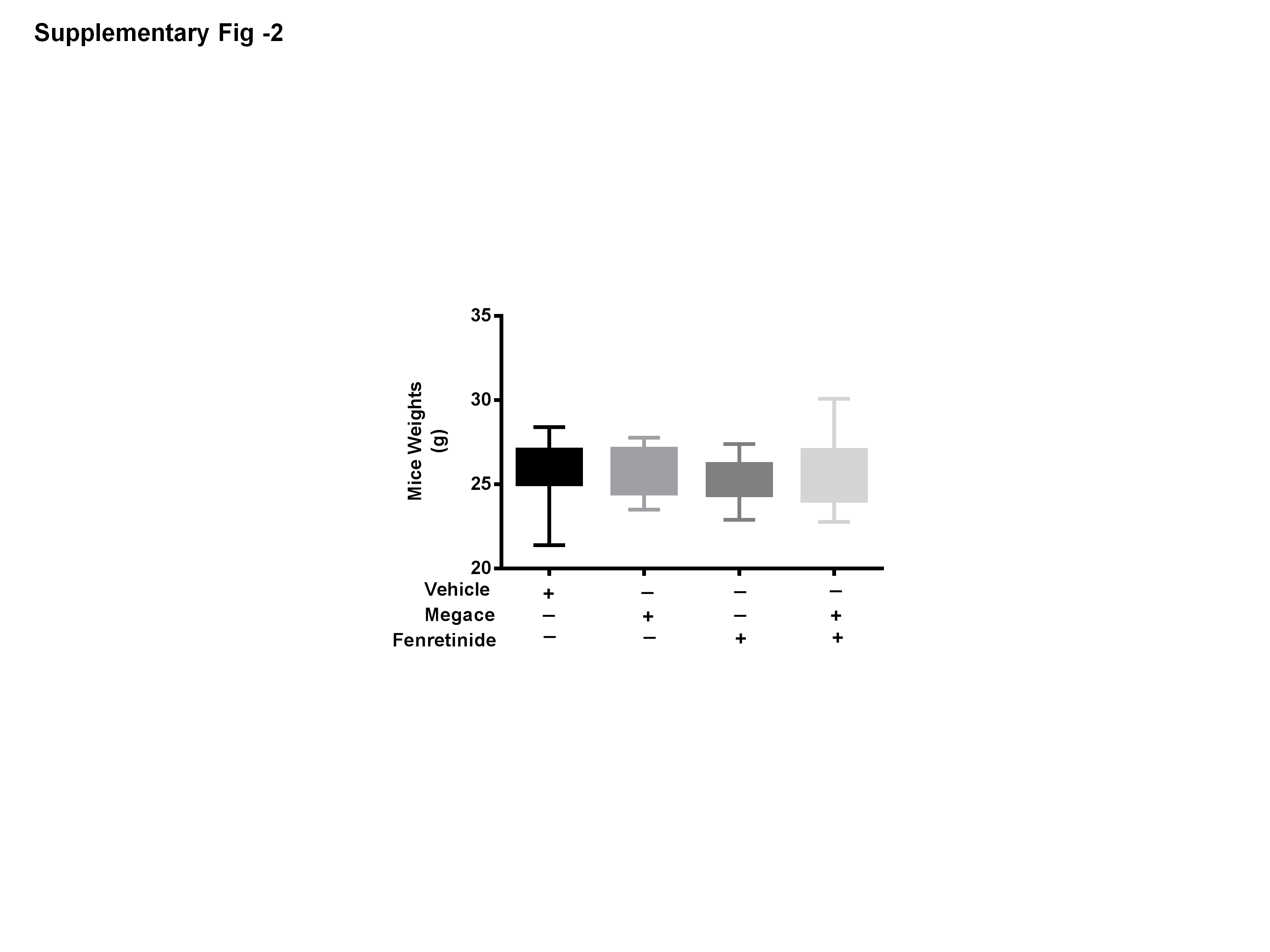

Supplement: Figure S2 — Treatments with either fenretinide or megace have no effect on mouse body weights. The body weights were measured from Vehicle treated or drug treated groups of mice twice a week during the whole experiment rime. (TIF) [file pone.0110410.s002.tif]
